# Repolarization of tumor infiltrating macrophages and increased survival in mouse primary CNS lymphomas after XPO1 and BTK inhibition

**DOI:** 10.1007/s11060-020-03580-y

**Published:** 2020-07-20

**Authors:** Isabel Jiménez, Júlia Carabia, Sabela Bobillo, Carles Palacio, Pau Abrisqueta, Carlota Pagès, Juan C. Nieto, Josep Castellví, Francisco Martínez-Ricarte, Lourdes Escoda, Cristóbal Perla, Dennis H. Céspedes Torrez, Joan Boix, Noelia Purroy, Lluís Puigdefàbregas, Joan Seoane, Francesc Bosch, Marta Crespo

**Affiliations:** 1 Experimental Hematology, Vall d’Hebron Institute of Oncology (VHIO), Vall d’Hebron Barcelona Hospital Campus, C/Natzaret, 115-117, Barcelona, 08035 Spain; 2 Department of Medicine, Universitat Autònoma de Barcelona, Bellaterra, 08193 Spain; 3 Department of Hematology, Experimental Hematology, Vall d’Hebron Hospital Universitari, Vall d’Hebron Institute of Oncology (VHIO), Vall d’Hebron Barcelona Hospital Campus, Passeig Vall d’Hebron 119-129, Barcelona, 08035 Spain; 4 Department of Pathology, Vall d’Hebron Hospital Universitari, Vall d’Hebron Barcelona Hospital Campus, Passeig Vall d’Hebron 119-129, Barcelona, 08035 Spain; 5 Department of Neurosurgery, Vall d’Hebron Hospital Universitari, Vall d’Hebron Barcelona Hospital Campus, Passeig Vall d’Hebron 119-129, Barcelona, 08035 Spain; 6 Department of Hematology, Hospital Universitari Joan XXIII, Tarragona, Spain; 7 Department of Neurosurgery, Hospital Universitari Joan XXIII, Tarragona, Spain; 8 Translational Research Program, Vall d’Hebron Institute of Oncology (VHIO), Vall d’Hebron Barcelona Hospital Campus, C/Natzaret, 115-117, 08035 Barcelona, Spain; 9 Institució Catalana de Recerca I Estudis Avançats (ICREA), CIBERONC, Barcelona, Spain; 10 Department of Medical Oncology, Dana-Farber Cancer Institute, Boston, Massachusetts and Broad Institute of MIT and Harvard, Cambridge, MA UK

**Keywords:** PCNSL, XPO1, BTK, Innate immune system

## Abstract

**Background:**

Patients diagnosed with primary central nervous system lymphoma (PCNSL) often face dismal outcomes due to the limited availability of therapeutic options. PCNSL cells frequently have deregulated B-cell receptor (BCR) signaling, but clinical responses to its inhibition using ibrutinib have been brief. In this regard, blocking nuclear export by using selinexor, which covalently binds to XPO1, can also inhibit BCR signaling. Selinexor crosses the blood–brain barrier and was recently shown to have clinical activity in a patient with refractory diffuse large B-cell lymphoma in the CNS. We studied selinexor alone or in combination with ibrutinib in pre-clinical mouse models of PCNSL.

**Methods:**

Orthotopic xenograft models were established by injecting lymphoma cells into the brain parenchyma of athymic mice. Tumor growth was monitored by bioluminescence. Malignant cells and macrophages were studied by immunohistochemistry and flow cytometry.

**Results:**

Selinexor blocked tumor growth and prolonged survival in a bioluminescent mouse model, while its combination with ibrutinib further increased survival. CNS lymphoma in mice was infiltrated by tumor-promoting M2-like macrophages expressing PD-1 and SIRPα. Interestingly, treatment with selinexor and ibrutinib favored an anti-tumoral immune response by shifting polarization toward inflammatory M1-like and diminishing PD-1 and SIRPα expression in the remaining tumor-promoting M2-like macrophages.

**Conclusions:**

These data highlight the pathogenic role of the innate immune microenvironment in PCNSL and provide pre-clinical evidence for the development of selinexor and ibrutinib as a new promising therapeutic option with cytotoxic and immunomodulatory potential.

**Electronic supplementary material:**

The online version of this article (10.1007/s11060-020-03580-y) contains supplementary material, which is available to authorized users.

## Background

Primary central nervous system lymphoma (PCNSL) is a rare and aggressive non-Hodgkin lymphoma (NHL) localized to the CNS in the absence of systemic involvement that represents around 4% of all brain tumors and 4 to 6% of all extranodal lymphomas [1]. Approximately 95% of PCNSL are classified as activated B-cell diffuse large B-cell lymphoma (ABC-DLBCL) based on histopathology, gene expression and mutational landscape [2]. Current treatment options for PCNSL include high doses of chemotherapy able to cross the blood–brain barrier (BBB) combined with anti-CD20 monoclonal antibodies and the addition of whole brain radiation in some settings; also, autologous stem cell transplantation is considered for young patients. Patients diagnosed with PCNSL respond poorly to the available treatments and often face dismal outcomes, especially in the relapsed setting, with an estimated overall survival of 30% at 5 years [3]. This notion of the poor prognosis of PCNSL can be explained by particular biological characteristics of the tumor. First, PCNSL are characterized by a high frequency of concomitant MYD88 and CD79B mutations [4] along with lesions related to B-cell development and function (e.g. BLIMP1), and the NF-κB pathway (e.g. CARD11 or TBL1XR1). The involvement of the BCR signaling in PCNSL has prompted the use of the BTK inhibitor ibrutinib, that, although it can cross the BBB [5], achieves wide but short duration responses [6–8]. In addition, PCNSL develop in a special microenvironment of unique immune surveillance, which could contribute to an inefficient response of the immune system against lymphoma cells. In this regard, the few reports examining the tumor-infiltrating immune microenvironment show that it is mainly composed by macrophages and by T-cells to a lesser extent [9–13]. Also, an intriguing high proportion of PCNSL have genetic lesions that potentially avoid being recognized by T-cells, namely HLA loses and PD-L1/2 amplifications found in up to 80% of patients [14]. Finally, the poor prognosis can also be explained by the diminished capacity of some drugs to cross the BBB. Selinexor (KPT-330), a BBB permeable small molecule [15], is a Selective Inhibitor of Nuclear Export (SINE) compound that binds to the cargo binding pocket of XPO1 (exportin-1/CRM1) and inhibits its activity. This results in the nuclear accumulation of tumor suppressor proteins and cell cycle regulators together with the activation of tumor suppressor proteins, which translates in cell cycle arrest and specific anti-cancer activity across a wide range of hematological and solid malignancies [16]. In July 2019, selinexor was approved by the FDA to treat patients with multiple myeloma while in May 2020 it was approved for systemic relapsed/refractory DLBCL after positive results in a phase IIb trial [17]. Also, the ability of selinexor to inhibit both the BCR and the NF-κB signaling pathways makes this drug interesting for studies in NHL [16, 18]. Recently, in a clinical case study, selinexor was reported to inhibit refractory DLBCL with CNS involvement [19]. In order to provide a pre-clinical rationale for the design of new therapeutic strategies for patients diagnosed with PCNSL, herein we evaluate the role of XPO1 and BTK inhibition in intracerebral xenograft murine models, focusing on malignant cells and the innate immune microenvironment.

## Materials and methods

### In vivo modeling of PCNSL

All animal experiments were approved by the local Ethical Committee for the Use of Experimental Animals. Detailed methods including treatment schedules can be found in *Supplementary information*. Briefly, brains of eight-week-old athymic female mice were injected with OCI-Ly10 cells stably transfected with luciferase, as previously reported [20]. Tumor growth was monitored by bioluminescence imaging (BLI) using IVIS® Spectrum system and Living Image software (PerkinElmer).

Patient derived xenograft (PDX) model was established by intracerebral injection of human lymphoma cells isolated from a brain biopsy in eight-week-old NOD-SCID-γ (NSG) female mice. Next, expanded CD19^+^ tumor cells were inoculated into the brain parenchyma of eight-week-old athymic female mice as specified above. Human tumor sample was obtained from a patient diagnosed with PCNSL at Hospital Universitari Joan XIII, Tarragona (Spain) after approval from the local Clinical Research Ethics Committee according to the principles of the Declaration of Helsinki and obtaining written informed consent from the patient.

### Flow cytometry and immunohistochemistry (IHC) analysis

Mice brains were collected in cold RPMI-1640 medium immediately after euthanasia and the two hemispheres were separated with a razor blade. One hemisphere was used for IHC and the other one was processed for flow cytometry. Detailed methods can be found in *Supplementary information*.

### Statistical analysis

Results are expressed as the mean ± standard error of the mean (SEM) of at least four independent experiments or subjects. The statistically significant differences between groups were analyzed using the Mann–Whitney test or one or two-way ANOVA, and P < 0.05 was considered significant. Detailed methods can be found in *Supplementary information*.

## Results

### DLBCL cell lines have equivalent sensitivity to selinexor regardless of their cell of origin (COO)

**Fig. 1:**
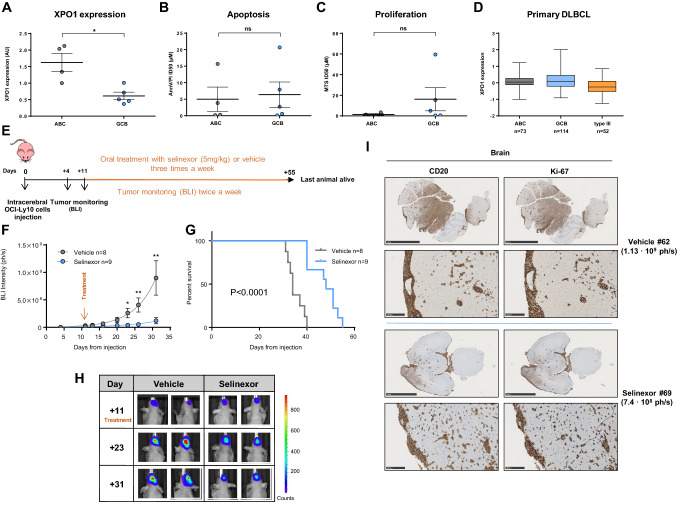
In vitro and in vivo effects of selinexor in PCNSL models. **a** XPO1 relative expression by QRT-PCR. Cells were treated with increasing doses of selinexor or vehicle (1% DMSO) for 96 h and viability and proliferation was determined by Annexin-V-PI exclusion (**b**) or MTS method (**c**). **d** Relative XPO1 expression in DLBCL patients, using public data from ref [24]. **e** Scheme representing mice treatment and monitoring. **f** Tumor size as measured by BLI in mice treated with vehicle (n = 8) or selinexor (n = 9). Data is shown until day 31, last day when all animals were still alive. Two-way ANOVA analysis (P = 0.0002). Asterisks indicate the result of Mann–Whitney test at different time points. (*P < 0.05, **P < 0.01, ***P < 0.001. Graphs show mean ± SEM) (**g**) Survival curves and (**h**) representative BLI images of the CNS tumors. **i** IHC analysis showing expression of CD20 and Ki-67 in representative mice brain parenchyma and meninges. The bars represent 5 mm in top panels and 250 µm in bottom panels. ID50: inhibitory dose 50. ABC: activated-B cell. GCB: germinal center B-cell. BLI: bioluminescence imaging. Ph/s: photons per second. (*P < 0.05, **P < 0.01, Mann–Whitney test. Graphs show mean ± SEM)

ABC-DLBCL relies heavily on NF-κB signaling and shows chronic BCR activation that is needed for survival, which translates into differential sensitivity to drugs targeting these pathways between ABC and GCB DLBCL cases [21, 22]. Since increased expression of XPO1 has been related to resistance to chemotherapy and worse prognosis in different neoplasias [23], we studied the potential relationship between expression of XPO1 and sensitivity to selinexor in DLBCL cell lines. Although mRNA expression of XPO1 was significantly higher in ABC-DLBCL cell lines (Fig. 1a), we did not find differential in vitro sensitivity to selinexor according to COO (Fig. 1b, c). Finally, we interrogated the publicly available data on gene expression of primary DLBCL cases [24] and we did not observe any association between the COO and the expression of XPO1 (Fig. 1d).

### Selinexor blocks tumor growth and prolongs survival in a bioluminescent orthotopic mouse model of PCNSL.

We next assessed the role of XPO1 inhibition in PCNSL using an intracerebral orthotopic xenograft murine model established by stereotactic injection of the luciferase-expressing OCI-Ly10 cell line into the cerebral parenchyma of nude athymic mice. OCI-Ly10 cell line was selected because it is derived from a patient diagnosed with ABC-DLBCL and its genetic profile includes mutations in *MYD88* (L265P) and *CD79A* (c. 4275_4316del) genes [20] (further verified in house), frequent in PCNSL [4]. Additionally, OCI-Ly10 cells have successfully been used before in a PCNSL xenograft model in athymic mice for pre-clinical studies [20]. Tumoral growth was monitored using IVIS-Spectrum bioluminescence measurement. Eleven days after the injection of cells, all animals had developed detectable tumors restricted to the CNS and were randomly distributed into treatment or vehicle experimental groups (vehicle: n = 8, mean radiance = 1.16·10^7^ ph/s ± 0.615·10^7^; treatment: n = 9, mean radiance = 2.32·10^7^ ph/s ± 1.86·10^7^). Mice were dosed with 5 mg/kg of selinexor or vehicle via oral gavage three times a week and subsequently, in order to non-invasively monitor the tumor growth, bioluminescence was assessed twice a week (Fig. 1e). Dose was selected based on previous pre-clinical data in mouse models of different neoplasias [25]. Treated mice showed a significantly slower increase in bioluminescence signal along time (two-way ANOVA: p = 0.0002; Fig. 1f) indicating that the treatment with selinexor was able to notably slow down tumor growth. Specific time-point analysis showed that differences were significant as soon as 12 days after start of treatment (day 23 after injection: vehicle mean radiance 2.61·10^8^ ph/s ± 8.64·10^7^ vs. 3.73·10^7^ ph/s ± 1.9·10^7^ in selinexor; p = 0.011) while differences peaked at day 20 after treatment (day 31 after injection: 8.98·10^8^ ph/s ± 3.13·10^8^ in vehicle vs. 1.19·10^8^ ph/s ± 5.58·10^7^ in selinexor group; p = 0.0037; Fig. 1f, representative cases can be seen in Fig. 1h). The blockage of intracerebral lymphoma growth induced by selinexor translated into a significantly increased survival, with a median survival of 48 days in the treatment group compared to 34 days in the vehicle group (p < 0.0001; Fig. 1g). At final point, histopathological analysis showed multifocal and infiltrative tumors affecting cerebral parenchyma and meninges of both cerebral hemispheres. Cells were highly proliferative (Ki-67 100%), CD20-positive and were often found in the perivascular space resembling human PCNSL histology. Remarkably, infiltration was observed in both hemispheres, showing no preference for the right hemisphere, where the original inoculation of malignant cells was performed. Also, we did not observe variations in CD20 intensity among mice or within different areas of the same brain (representative cases shown at Fig. 1i and Supplemental Figure S1).

### The combination of selinexor and ibrutinib synergizes in vitro in DLBCL cell lines and increases survival of mice with CNS lymphoma

The high frequency of molecular alterations in components of the BCR pathway can in part explain the response to BCR inhibitors in PCNSL. In this regard, ibrutinib in monotherapy in patients diagnosed with relapsed or refractory PCNSL achieves higher response rates compared to systemic DLBCL, however, the duration of the response is brief [5–7]. Alongside this, SINE compounds have also been shown to inhibit BCR signaling by downregulating the protein expression of BTK via enforced IκB nuclear retention in primary cells from patients with chronic lymphocytic leukemia (CLL) [16]. Moreover, the combination of selinexor and ibrutinib has shown in vitro synergism in CLL cells [18]. Accordingly, we observed reduced BCR signaling after treatment of OCI-Ly10 cells with selinexor and ibrutinib (Supplementary Figure S2A), as well as reduced BTK expression after 48 h of treatment with selinexor (Supplementary Figure S2B). Against this background, we hypothesized that combining XPO1 and BTK inhibition in PCNSL would have a synergistic therapeutic effect in our models. Firstly we treated a panel of cell lines in vitro with increasing doses of both drugs and analyzed apoptosis after 96 h. In three out of four ABC-DLBCL cell lines we observed a strong synergism between the two compounds (Supplementary Figure S2C); remarkably, treatment with selinexor sensitized GCB-SUDHL4 cells to ibrutinib, as shown by the combination index values indicating strong synergism between the two drugs (CI) (Supplementary Figure S2C, right panel).

**Fig. 2:**
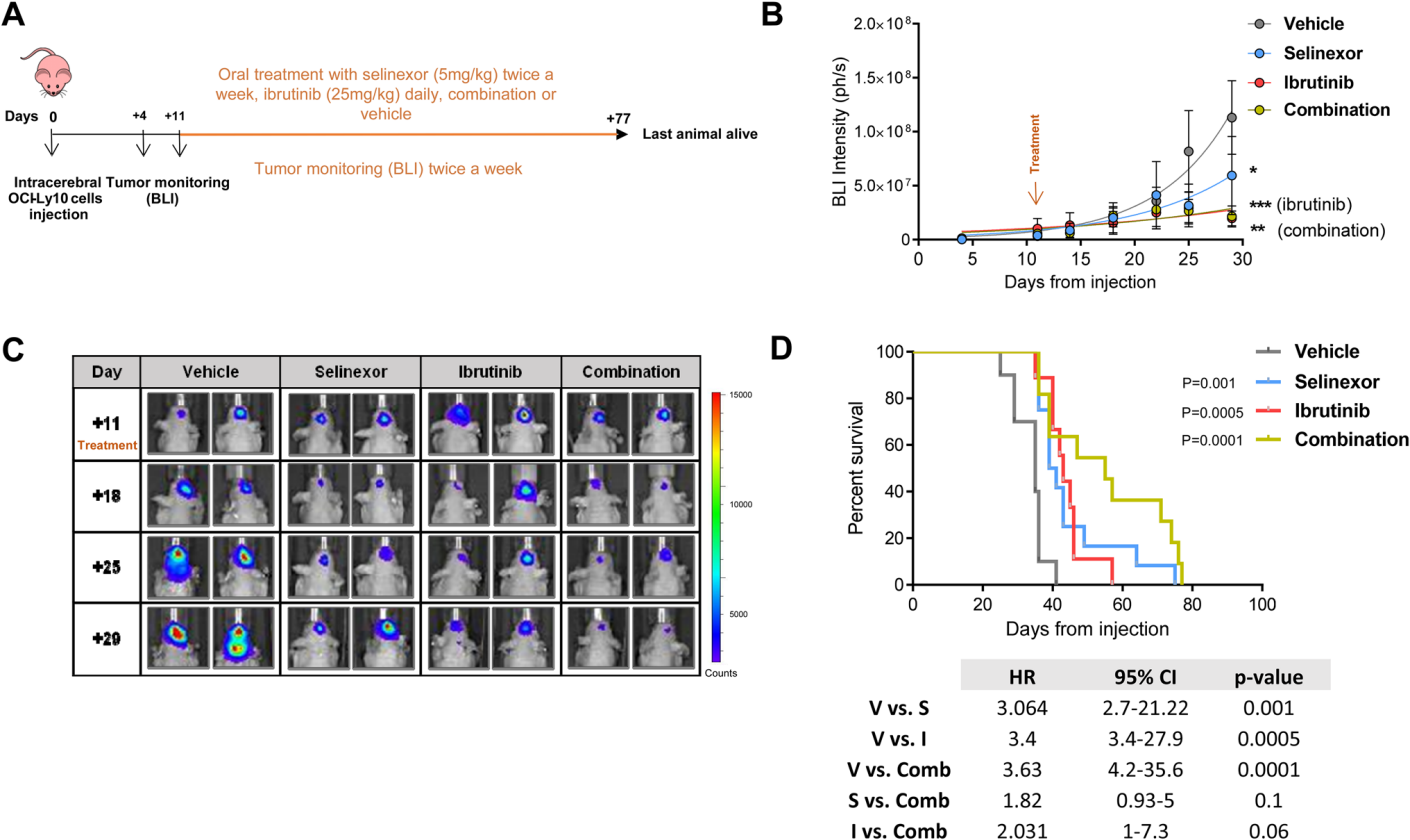
Treatment with selinexor and ibrutinib further increases survival of mice with CNS lymphoma. **a** Scheme representing mice treatment and monitoring. **b** Tumor size as measured by BLI intensity. Data is shown until day 29, last day when all animals were still alive. (*P < 0.05, **P < 0.01, ***P < 0.001, Mann–Whitney test. Graphs show mean ± SEM). **c** Representative BLI images in mice from every treatment arm. **d** Survival curves of mice in the four treatment groups. Survival curves were generated using the Kaplan and Meier method, and statistically compared by the log-rank test. *HR* hazard ratio, *CI* confidence interval, *BLI* bioluminescence imaging

We next sought to elucidate whether the synergy observed in vitro could be translated in vivo. Importantly, while ibrutinib is mainly metabolized by cytochrome P450, the metabolism of selinexor is independent of it, therefore it is unlikely that their co-administration could result in any effects on the exposure for the other drug [25, 26]. By using the same animal model described above, mice were distributed into the following four groups and started therapy 11 days after intracerebral injection of lymphoma cells: selinexor monotherapy (5 mg/kg twice a week via oral gavage, n = 12, mean radiance = 3.95·10^6^ ph/s), ibrutinib monotherapy (25 mg/kg daily in drinking water, n = 9, mean radiance = 1.02·10^7^ ph/s), combination therapy (n = 11, mean radiance = 1.02·10^7^ ph/s) and vehicle (n = 9, mean radiance = 3.21·10^6^ ph/s). Selinexor dose was adjusted (from three times a week to twice a week) in order to prevent potential toxicity of the drug combination, while ibrutinib dose was based on previous experience in CLL preclinical models [27] (Fig. 2a). Compared to vehicle, all three treatment regimens induced an equivalent significant effect in tumor growth kinetics in terms of decreased growth rate (Fig. 2b and c). Interestingly, the combination increased the survival of mice compared to vehicle, whereas there was no significant difference between ibrutinib and selinexor alone. Although the median survival increased up to 55 days, the survival curve of the mice treated with the combination was not statistically different from the ones from mice treated with the individual treatments (median survival of mice treated with vehicle: 35 days vs. survival for mice treated with selinexor: 40 days, p = 0.001; vehicle vs. ibrutinib, 43 days, p = 0.0005; vehicle vs. combination, 55 days, p = 0.0001; Fig. 2d).

### CNS lymphoma is infiltrated by tumor-promoting M2-like macrophages expressing PD-1 and SIRPα

**Fig. 3:**
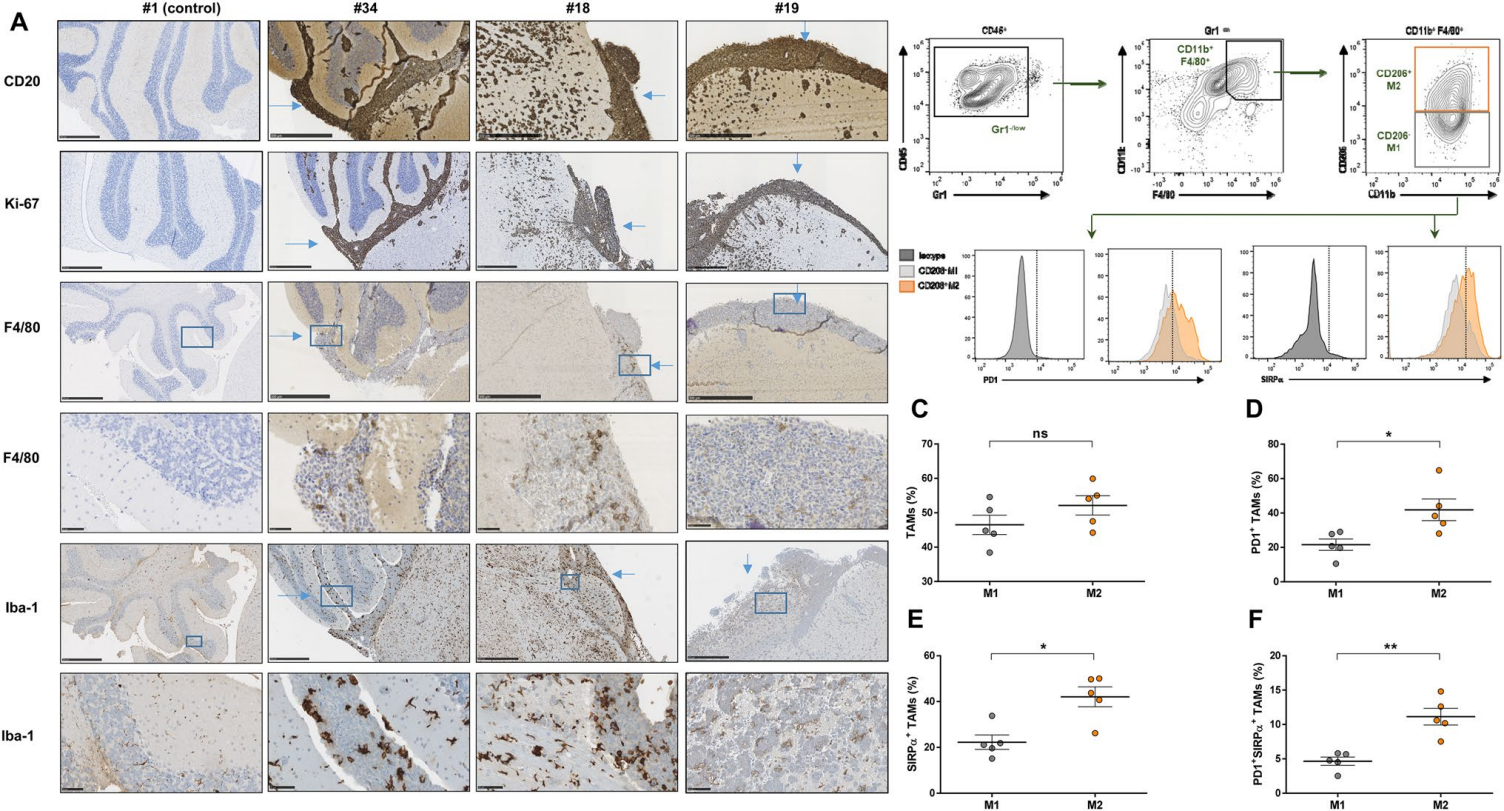
OCI-Ly10 CNS lymphomas are infiltrated by innate immune cells. **a** Representative IHC images from brains obtained from three mice inoculated with OCI-Ly10 cells (24 days after injection). The bar represents 500 µm, except for fourth and last rows (50 µm). **b** Gating strategy for the analysis of TAMs. Percentage of macrophages (M1/M2) (**c**) expressing PD-1 (**d**), SIRPα (**e**) and co-expressing both (**f**)

Analysis of the tumor-infiltrating immune microenvironment has shown that tumoral cells in PCNSL are accompanied by tumor-associated macrophages (TAMs) and T-cells to less extend, which is related to bad prognosis. [9–13] Remarkably, TAMs in mouse and human colorectal cancer have been recently described to express the immune checkpoint PD-1 and to recover their potential to phagocyte tumoral cells when PD-1 is blocked [28]. To conduct an interactive study of the infiltrating innate immune cells and PCNSL, we inoculated OCI-Ly10 cells into the brain parenchyma of nude athymic mice, an experimental in vivo model that has been previously successfully used to study the modulation of the innate immune response against PCNSL [20, 29]. Brains were harvested after 24 days of cell injection and further processed for subsequent analysis. Histopathological analysis showed that tumors encompassing both cerebral hemispheres were infiltrated by macrophages expressing the surface glycoprotein F4/80, mainly in the meninges but also in the cerebral parenchyma; notably, F4/80-positive macrophages were completely absent in the areas of the brain that were not invaded by tumoral cells (Figs. 3a and Supplemental figure S1) as well as in healthy brains from control mice (Fig. 3a). Iba-1 staining further identified microglial cells and TAMs, which showed an amoeboid morphology when interacting with tumoral cells, consistent with an active state (Fig. 3a) [30]. TAMs can be polarized towards a pro-inflammatory (M1) or a tumor-promoting (M2) state, depending on microenvironment and external stimuli [31]. By flow cytometry, we analyzed the proportion of M1 and M2 TAMs and their expression of immune checkpoints in brains from mice with PCNSL. First, we observed that TAMs were evenly distributed between M1 and M2 (Fig. 3c). Of note, TAMs expressed PD-1, mainly the tumor promoting M2 subset (Fig. 3d). This suggests that the direct interaction of M2 macrophages with the tumor triggers the upregulation of PD-1 and thus impairs their phagocytic capacity, as has been recently discovered in an analogous role to tumor-infiltrating T-cells using both immunocompetent syngeneic and athymic xenograft mouse models [28]. SIRPα is a well described regulatory checkpoint on macrophages, its interaction with CD47 on malignant cells hampering the phagocytosis by macrophages [32]. Herein we observed that SIRPα was also preferentially expressed by M2 TAMs (Fig. 3e) and that the co-expression of PD-1 and SIRPα was also higher in the M2 subset (Fig. 3f), pointing out towards a severe inhibition of macrophage activity in CNSL.

**Fig. 4:**
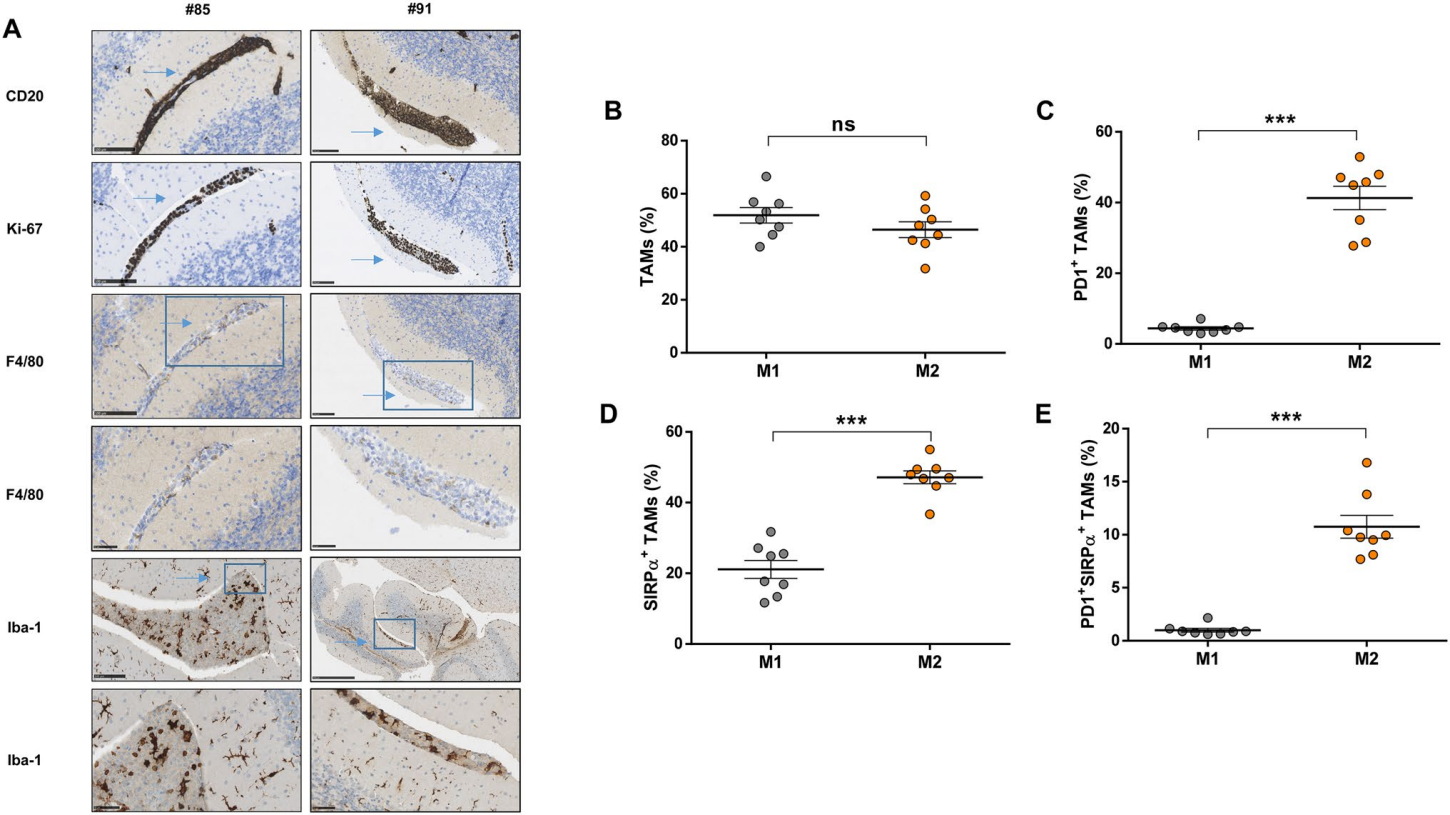
PDX CNS lymphomas are infiltrated by innate immune cells. **a** Representative IHC images from brains obtained from two mice inoculated with patient-derived PCNSL cells (18 days after injection). The bar represents 100 µm except for the four last rows (50 µm). Percentage of macrophages (M1/M2) (**b**) expressing PD-1 (**c**), SIRPα (**d**) and co-expressing both (**e**). (*P < 0.05, **P < 0.01, ***P < 0.001, Mann–Whitney test. Graphs show mean ± SEM)

The response of the innate immune system to PCNSL cells derived from a patient was further analyzed. For that, we developed an orthotopic PDX model using NSG mice to initially expand the freshly obtained primary malignant cells, as previously described by Rubenstein et al. and following the detailed protocol described in Supplementary methods [33] Next, we inoculated 2·10^5^ lymphoma cells into the brain parenchyma of nude athymic mice [34]. Since the median survival of this mouse model was 22 days, infiltration by immune cells was analyzed after 18 days of tumor injection allowing infiltration by innate immune cells. In this model, TAMs were also found only amongst tumoral cells (Figs. 4a and Supplemental Figure S3) as assessed by IHC. TAMs from the PDX model displayed an immunophenotypic profile resembling the one found in TAMs from the cell line xenograft model. Along this line, a similar proportion of M1 and M2 (Fig. 4b) and a more frequent expression of PD-1 and SIRPα in M2 tumor-promoting macrophages was observed (Fig. 4c, d, e). In contrast to the OCI-Ly10 model, patient-derived PCNSL cells did express the SIRPα ligand CD47 (97.61% of CD20 cells ± 0.62).

### Treatment with selinexor and ibrutinib favors TAM polarization toward pro-inflammatory M1-like and diminishes PD-1 and SIRPα expression in M2-like TAMs

**Fig. 5:**
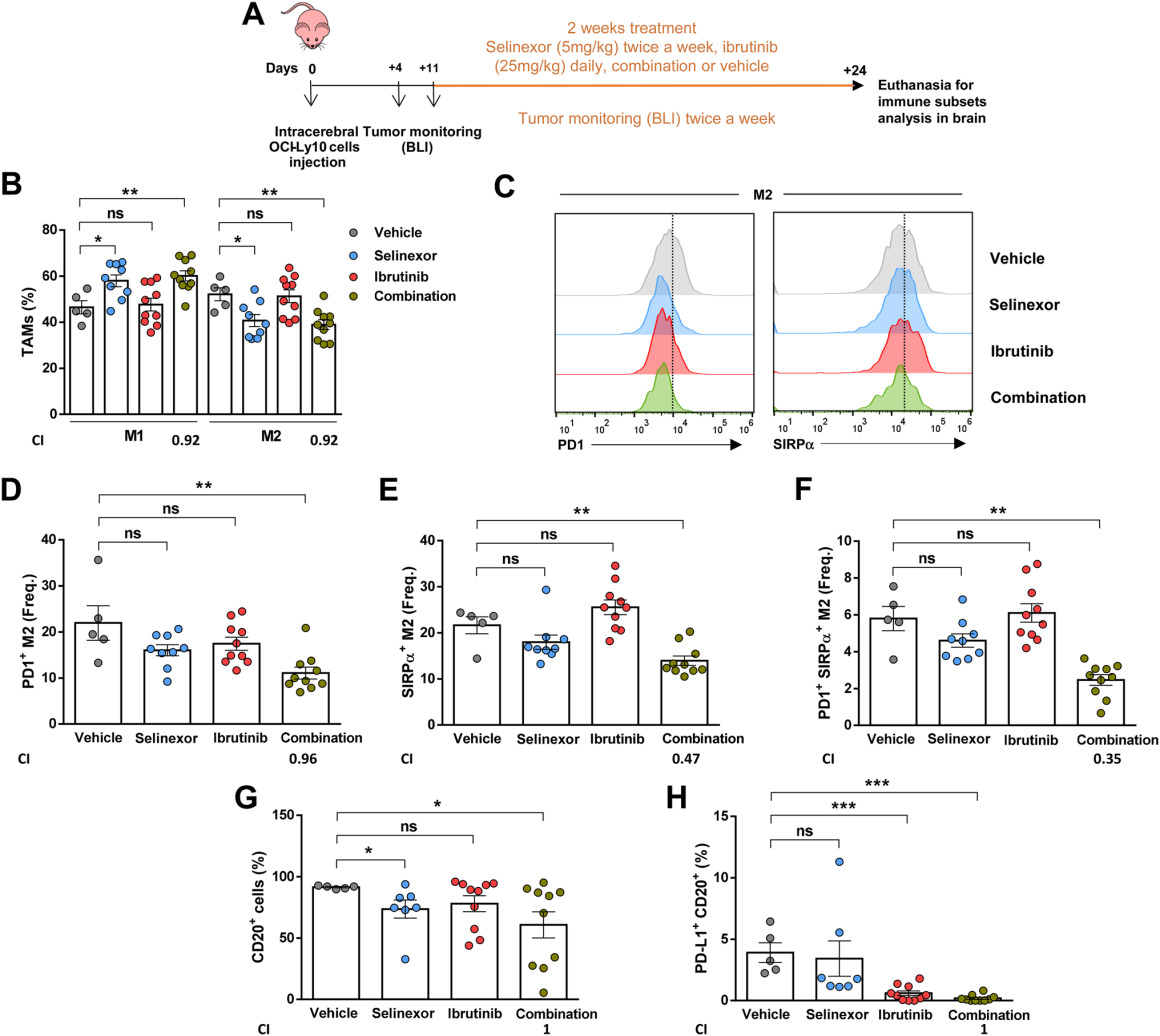
Treatment with selinexor and ibrutinib favors M1-like response in tumor-associated macrophages in OCI-Ly10-derived CNS lymphomas. **a** Scheme representing mice treatment and monitoring. **b** Percentage of M1 and M2 TAMs by flow cytometry. **c** Histograms of PD1^+^ M2 and SIRPα^+^ M2 of one representative mouse from each group. Frequency of M2 macrophages that express PD-1 (**d**), SIRPα (**e**) or co-express both markers (**f**). **g** Percentage of CD20^+^ cells in the brains from mice treated for two weeks. **h** Percentage of CD20^+^ malignant cells expressing PD-L1 in the different treatment groups. (*P < 0.05, **P < 0.01, ***P < 0.001, Mann–Whitney test. Graphs show mean ± SEM). *CI* combination index, *BLI* bioluminescence imaging

BTK protein has been shown to be crucial for tumor-promoting function of macrophages in different neoplasias, especially in CLL, where modulation of TAMs has been shown to be also a relevant mode of action of ibrutinib [35, 36]. Therefore, after showing that the combination of selinexor and ibrutinib restrains tumor growth and prolongs mice survival, and since both drugs are able to inhibit BTK, we hypothesized that these drugs could also cooperate to modify the innate immune response in PCNSL. In this regard, pre-clinical PCNSL models have previously demonstrated how immunomodulating drugs are able to shift macrophages polarization as well as have direct antitumoral effect. [20, 29] To test that, we treated mice bearing OCI-Ly10-CNS lymphomas with selinexor 5 mg/kg twice a week, ibrutinib 25 mg/kg daily or the combination of the two drugs for two weeks by oral gavage (Fig. 5a). We observed that selinexor and the combination shifted the M1/M2 ratio towards predominance of anti-tumoral M1 (Fig. 5b). Interestingly, while none of the individual treatments induced significant changes in the frequency of PD-1 or SIRPα-positive M2 macrophages, the drug combination significantly reduced the frequency of PD-1-positive, SIRPα-positive (Fig. 5c, d, e) and double-positive M2 macrophages (Fig. 5f). In agreement the (CI) that the reduction of the expression of PD-1, SIRPα and their co-expression was synergistic (CI < 1). This was accompanied by a reduction in PD-L1-expressing malignant cells (Fig. 5g, h) that was attributable to ibrutinib action since it was also observed under ibrutinib monotherapy.

**Fig. 6:**
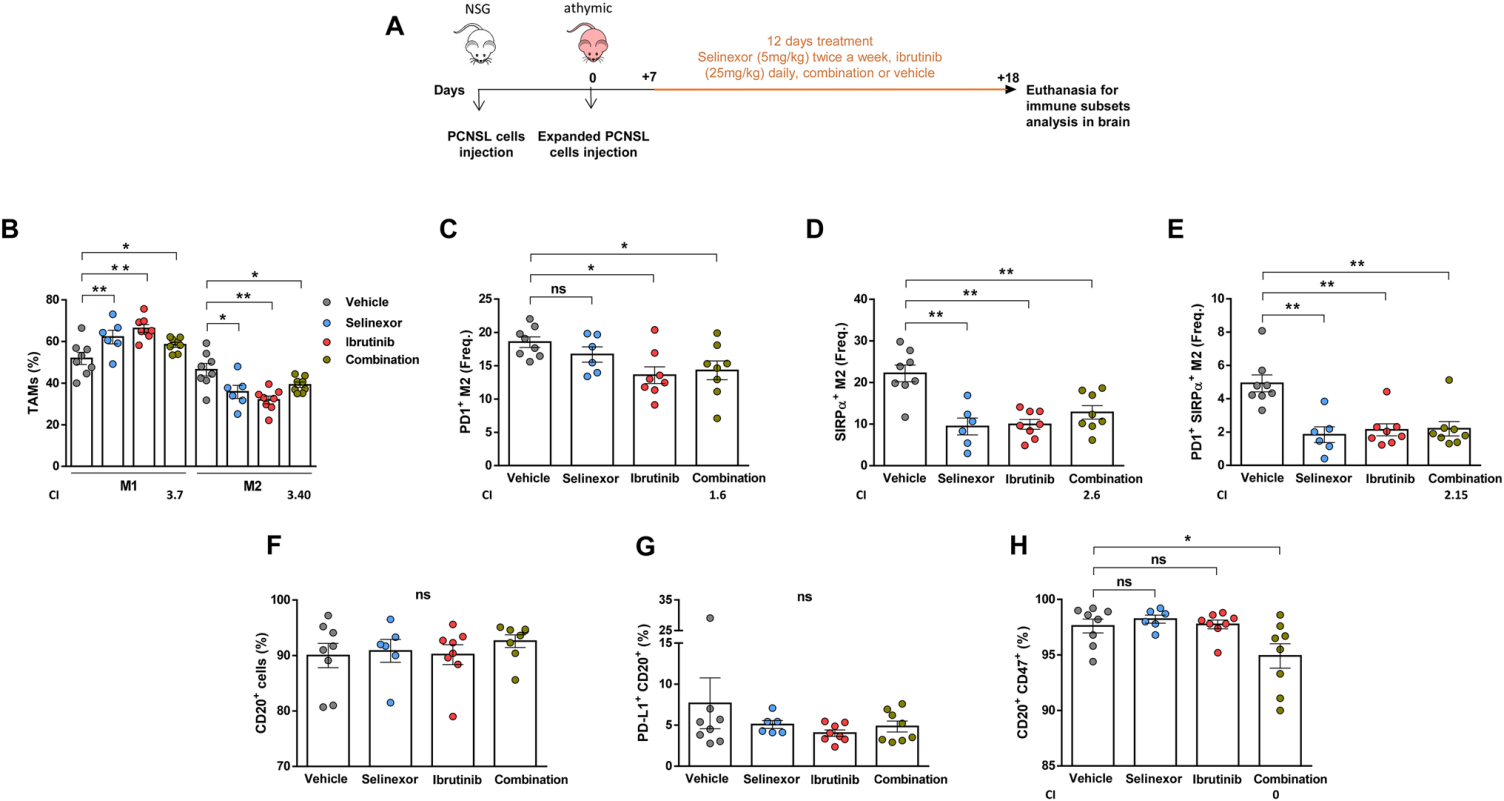
Treatment with selinexor and ibrutinib favors M1-like response in tumor-associated macrophages in CNS lymphoma PDXs. **a** Scheme representing mice treatment and monitoring. **b** Percentage of M1 and M2 TAMs by flow cytometry. Frequency of M2 macrophages that express PD-1 (**c**), SIRPα (**d**) or co-express both markers (e). **f** Percentage of CD20^+^ cells in the brains from mice. **g** Percentage of malignant cells CD20^+^ expressing PD-L1 in the different treatment groups. Percentage of malignant cells expressing CD47 (**h**) and co-expressing PD-L1 and CD47 (**i**). (*P < 0.05, **P < 0.01, ***P < 0.001, Mann–Whitney test. Graphs show mean ± SEM). *CI* combination index

In the PDX model, the study of immunomodulation was performed 18 days after cell injection preceded by 12 days of oral gavage treatment as described earlier (Fig. 6a). Both treatments alone or in combination were able to change the M1/M2 balance towards a more anti-tumoral or inflammatory response (Fig. 6b). Moreover, treatment with ibrutinib only or with the drug combination was able to diminish the frequency of PD-1-positive M2 macrophages (Fig. 6c). The frequency of SIRPα-positive M2 macrophages was also diminished by both individual treatments, as well as the double positive M2 cells (Fig. 6d and e). In this mouse model we did not observe any effect in the expression of PD-L1 by the malignant cells, while the percentage of malignant cells was also not affected by the short term treatment (Fig. 6f and g). Expression of CD47 by patient-derived PCNSL cells was significantly downregulated after treatment with the combination (Fig. 6h). Accordingly, CI calculations show that the combination did not improve upon individual treatments for any of the parameters except for the expression of CD47 on malignant cells. In order to identify direct immunomodulatory effects of selinexor and ibrutinib on human macrophages, we treated peripheral blood-derived macrophages in vitro with increasing doses of selinexor, ibrutinib or the combination for 30 min before inducing differentiation to M2 using macrophage colony-stimulating factor (M-CSF) and IL-10 (see *Supplementary information* for detailed methods). M2 macrophages derived from 8 healthy donors had a mean expression of PD-1 of 81.15% + /−8.8 and mean expression of SIRPα of 45.53% + /−9.3. Firstly, we made sure that the drugs did not affect survival of macrophages at the concentrations used (data not shown). Next, in agreement with what we observed in vivo, we observed downregulation of the expression of both PD-1 and SIRPα caused by individual drugs or the combination. (Supplementary Figures S4A, S4B and S4C). However, this did not translate into increased phagocytic activity (Supplementary Figure S4D).

Also, using the same experimental setting we analyzed the effect of selinexor, ibrutinib or the combination in interfering with M2 polarization by analyzing additional M1 and M2-like markers and IL-10 production. We found an increase in the expression of the activation and M1-like marker CD86 and a decrease in the M2-like marker CD163 as well as lower levels of PD-L1 and the anti-inflammatory cytokine IL-10 after treatment with selinexor and ibrutinib (Supplementary Figures S4E-H). However, we did not see any significant effect in the expression of CD206 or HLA-DR (Supplementary Figures S4I–J). In vitro modulation of additional surface markers and cytokines is consistent with the loss of pro-tumoral M2 properties after treatment with selinexor and ibrutinib.

Altogether these results indicate that the combination of selinexor and ibrutinib is able to block tumoral growth, to significantly increase the median survival of mice with PCNSL and to modulate the innate immune microenvironment towards a more anti-tumoral stage, likely reinvigorating the anti-tumoral phagocytic function of the tumor infiltrating macrophage population in vivo.

## Discussion

Blockage of XPO1-mediated nuclear transport using SINEs like selinexor has been shown to be an effective anti-neoplastic approach in a variety of malignancies. [17, 37, 38] XPO1 inhibition forces nuclear localization of tumor suppressors and also interferes with additional signaling pathways, including NF-κB and BCR, which are crucial for survival of malignant B cells in general and for PCNSL cells in particular. The clinical use of selinexor in lymphoma has been studied in a phase I trial studying patients diagnosed with relapsed/refractory NHL and a phase IIb study in patients with DLBCL [17], which has led to a recent approval by the FDA in such an adverse setting. Additionally, based on our pre-clinical experience, we recently used selinexor in a compassionate way for a patient diagnosed with DLBCL who developed an isolated CNS relapse after several lines of treatment. After a month of treatment a partial response was already observed while after 5 months of selinexor the patient remained asymptomatic and the MRI (magnetic resonance imaging) showed a complete resolution of the brain tumors [19]. Ibrutinib is also able to cross the BBB and is active against CNS lymphoma cells. In this setting, ibrutinib has been assayed alone [6, 7] or in combination with chemotherapy [5], showing high response rates but relatively short remissions, while other BTK inhibitors have showed similar efficacy [39]. Based on all these data, herein we proposed to combine selinexor with ibrutinib in models of PCNSL.

Exploitation of the immune response to a neoplastic process is currently a widespread strategy to treat cancer. To achieve this, different approaches are being pursued, specially focused on harnessing the anti-tumoral capacity of T lymphocytes via checkpoint inhibition [40]. Intriguingly, evading a T-cell mediated immune response seems to be a common feature of PCNSL since a high percentage of cases are affected by both MHC-I loss and/or PD-L1/2 amplification [14], and the infiltration by T lymphocytes is scarce while present [9–12]. However, some immunotherapies have already shown to be effective in PCNSL, such as anti-CD20 and, more recently, anti-PD-1 therapy, with both preclinical [41] and clinical evidences, although with only information for four patients, where responses lasted a median of 15 months [42]. In agreement, anti-PD-1 is highly effective in Hodgkin’s lymphoma [43] even though the expression of PD-1 on T-cells is heterogeneous and PD-L1/2 amplification and lack of MHC-I expression on tumoral cells are common, characteristics that should hamper a T-cell mediated response [44]. In this regard, a role for the innate immune system in the development of PCNSL is further supported by recent discovery of PD-1 expression in TAMs [28] and the fact that these immune cells have also been found to be suppressed by the MHC-I system in cancer cells, rendering malignant cells that downregulate MHC-I to avoid T cell surveillance exposed to macrophage phagocytosis [45]. Therefore, paralleling the few PCNSL patients treated with anti-PD-1 achieving a complete response, this effect may be related to a macrophage-mediated anti-tumoral effect after PD-1 pharmacological blockage. Supporting that, herein we describe the presence of brain PD-1-positive M2 macrophages in two orthotopic mouse models of PCNSL, including PDXs. The recognition of human malignant cells by mice macrophages has been previously demonstrated in mice models of PCNSL [20, 46] and other tumoral models such as colon cancer [28], pancreatic adenocarcinoma [47] and T-cell lymphoma [48]. TAMs in CNLS have been found to be supportive of the tumoral growth and related to prognosis of patients [9, 13]. Also, indoleamine 2,3 dioxygenase (IDO) and IL-10, which may be markers of macrophage infiltration, are related to prognosis or response to immunomodulatory therapy [9, 13, 49]. The observed expression of PD-1 and SIRPα by innate immune cells responding to and interacting with CNS lymphoma cells in vivo indicates that their anti-tumoral effect is partially impaired but also opens the opportunity to potentially target these cells by immunotherapies that aim at potentiating the autologous anti-tumoral immune response. In this regard, it has been previously shown how immunomodulation by pomalidome in mouse models of PCNSL results in reprogramming of M2 macrophages into M1 [20]. In the clinical setting, both pomalidomide and lenalidomide are showing preliminary therapeutic activity in a phase I study in patients diagnosed with PCNSL (combined with dexamethasone) [29]. Also, lenalidomide in combination with rituximab showed significant clinical activity in relapsed/refractory PCNSL patients [49, 50]. Combination therapies that not only directly attack the survival of malignant cells but also alter the immune function are therefore an interesting approach when aiming at achieving long lasting responses. In this regard, inhibiting BTK can have this double effect in B-cell malignancies, since BTK protein is not only involved in malignant B-cell survival but is also required for the tumor-promoting effect of macrophages [35, 36]. Taking this into account, we hypothesized that combining ibrutinib with selinexor would also be effective in harnessing the innate immune response mediated by TAMs in PCNSL. In fact, selinexor and ibrutinib combination treatment was able to not only increase mouse survival but to shift the innate immune response towards a more inflammatory phenotype, specifically defined by downregulation of PD-1 and SIRPα in M2 macrophages and increased proportion of M1 macrophages as well as modulation of additional M1 and M2-like properties consistent with loss of pro-tumoral M2 characteristics. Confirmation of these results and additional studies in the interaction of malignant cells and the immune system in PCNSL using different in vivo models, including syngeneic mice, is needed to further confirm the potential clinical value of the combination of selinexor and ibrutinib in patients diagnosed with PCNSL.

## Conclusions

Our results show that selinexor blocks tumor growth and prolongs survival in a bioluminescent mouse model, while its combination with ibrutinib further increases survival. Alongside this, treatment with this combination not only had a direct cytotoxic effect in malignant cells but also favored an anti-tumoral innate immune response by shifting polarization of tumor-infiltrating macrophages toward inflammatory M1 and diminishing PD-1 and SIRPα expression in the remaining tumor-promoting M2 macrophages, highlighting the pathogenic role of the innate immune microenvironment in PCNSL. Herein we provide pre-clinical evidence for the development of selinexor and ibrutinib as a new therapeutic option with cytotoxic and immunomodulatory potential for patients diagnosed with PCNSL, aiming at a durable response to improve the fatal prognosis of patients diagnosed with this disease.

## Electronic supplementary material

Below is the link to the electronic supplementary material.

Supplementary file1 (PPTX 48451 kb)

Supplementary file2 (DOCX 29 kb)

## Data Availability

The datasets used and/or analyzed and materials from the current study are available from the corresponding author on reasonable request.

## Abbreviations

PCNSL: Primary central nervous system lymphoma
BCR: B-cell receptor
NHL: Non-Hodgkin lymphoma
ABC-DLBCL: Activated B-cell diffuse large B-cell lymphoma
GCB-DLBCL: Germinal center B-cell diffuse large B-cell lymphoma
SINE: Selective inhibitor of nuclear export
BBB: Blood–brain barrier
BLI: Bioluminescence imaging
PDX: Patient derived xenograft
NSG: NOD-SCID-γ: non-obese diabetic- severe combined immune deficiency- interleukin 2 receptor gamma chain
IHC: Immunohistochemistry
SEM: Standard error of the mean
COO: Cell of origin
CLL: Chronic lymphocytic leukemia
TAM: Tumor-associated macrophages
M-CSF: Macrophage colony-stimulating factor
IDO: Indoleamine 2, 3 dioxygenase
MRI: Magnetic resonance imaging
CI: Combination index
