# Supplementary material for: Repolarization of tumor infiltrating macrophages and increased survival in mouse primary CNS lymphomas after XPO1 and BTK inhibition
Source: J Neurooncol. 2020 Jul 20;149(1):13–25. doi: 10.1007/s11060-020-03580-y (PMC7452938; doi:10.1007/s11060-020-03580-y)
Supplement: Supplementary file 1 — Supplementary file1 (PPTX 48451 kb) [file 11060_2020_3580_MOESM1_ESM.pptx]

## Slide 1
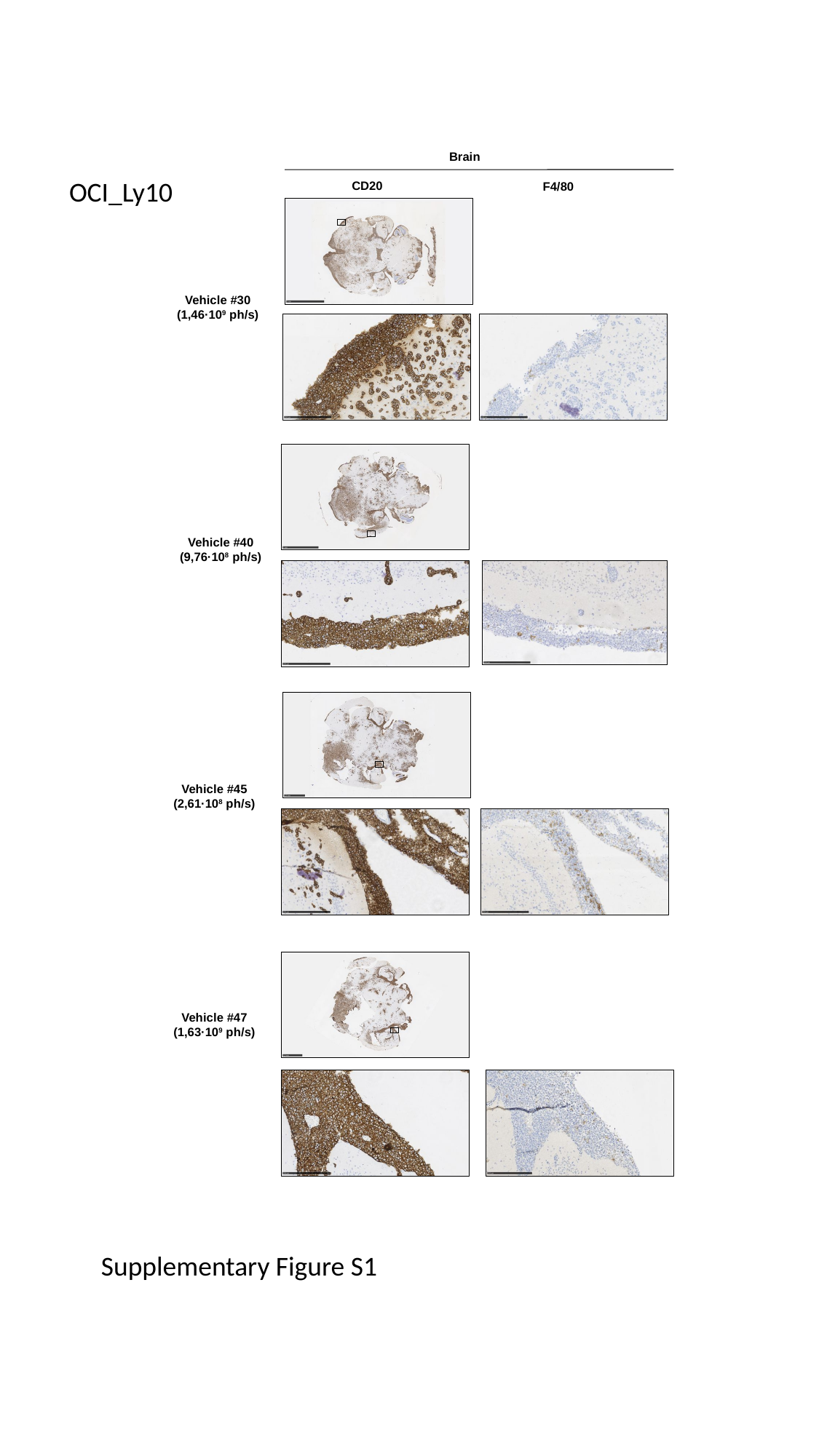

Brain
OCI_Ly10
CD20
F4/80
Vehicle #30
(1,46·109 ph/s)
Vehicle #40
(9,76·108 ph/s)
Vehicle #45
(2,61·108 ph/s)
Vehicle #47
(1,63·109 ph/s)
Supplementary Figure S1

## Slide 2
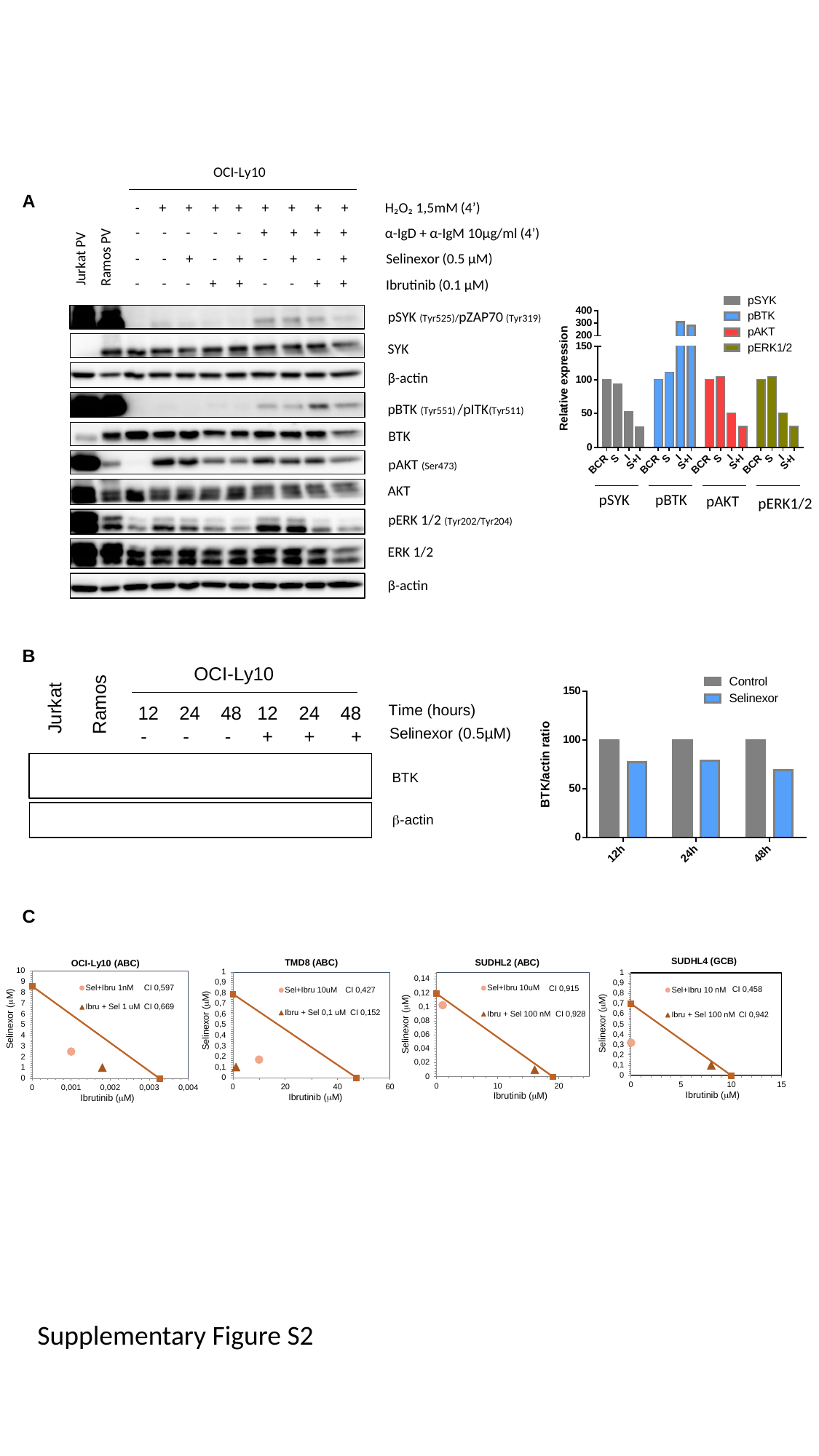

A
B
C
Supplementary Figure S2

## Slide 3
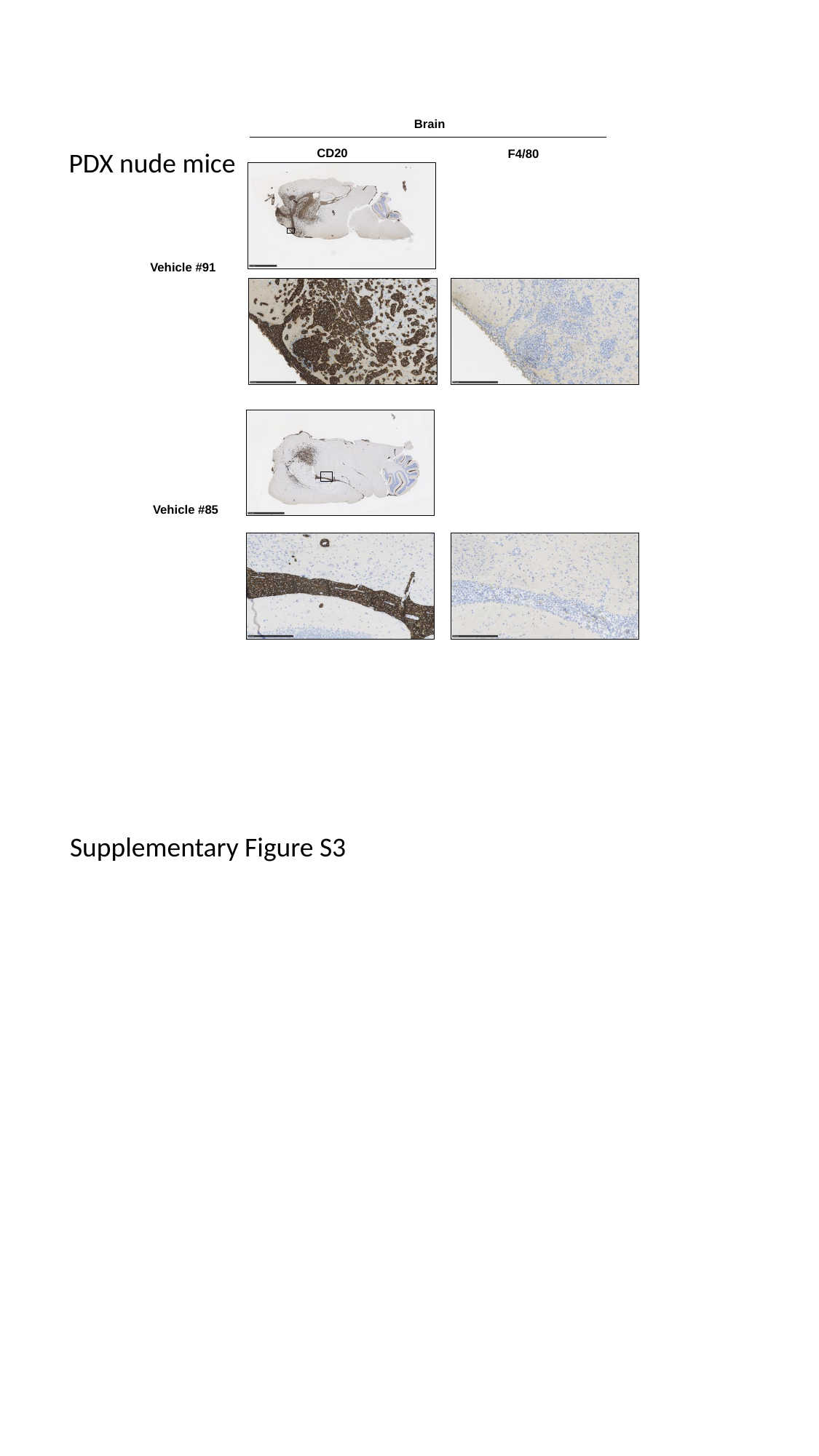

Brain
PDX nude mice
CD20
F4/80
Vehicle #91
Vehicle #85
Supplementary Figure S3

## Slide 4
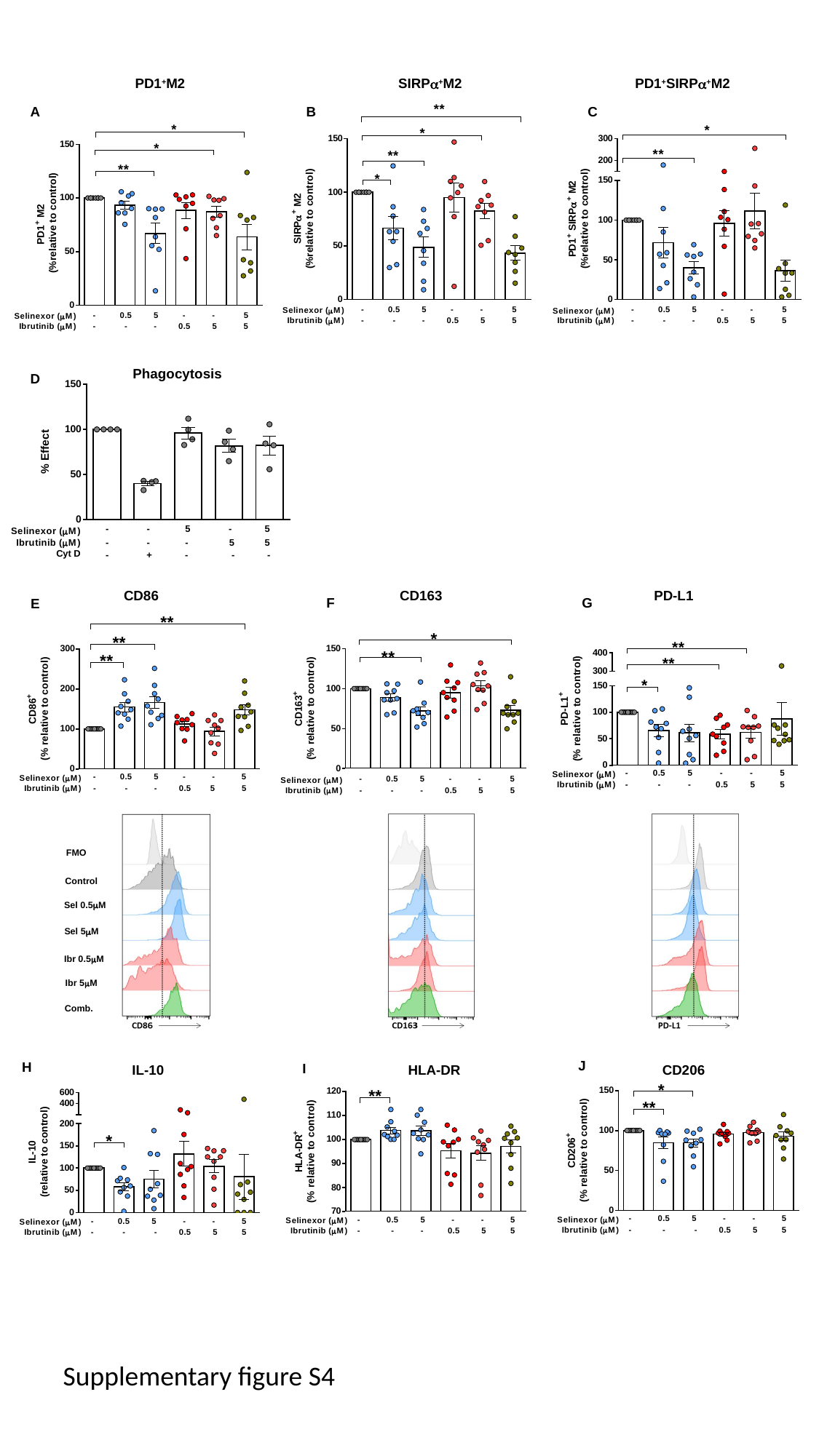

PD1+M2
SIRPa+M2
PD1+SIRPa+M2
A
B
C
Phagocytosis
D
CD86
CD163
PD-L1
G
F
E
FMO
Control
Sel 0.5mM
Sel 5mM
Ibr 0.5mM
Ibr 5mM
Comb.
J
H
I
IL-10
HLA-DR
CD206
Supplementary figure S4
