# Supplementary material for: Repolarization of tumor infiltrating macrophages and increased survival in mouse primary CNS lymphomas after XPO1 and BTK inhibition
Source: J Neurooncol. 2020 Jul 20;149(1):13–25. doi: 10.1007/s11060-020-03580-y (PMC7452938; doi:10.1007/s11060-020-03580-y)
Supplement: Supplementary file 2 — Supplementary file2 (DOCX 29 kb) [file 11060_2020_3580_MOESM2_ESM.docx]

Supplementary information for Jiménez et al ‘Repolarization of tumor infiltrating macrophages and increased survival in mouse primary CNS lymphomas after XPO1 and BTK inhibition’

**Supplementary methods**

**Reagents**

Selinexor was kindly provided by Karyopharm. Ibrutinib was kindly provided by Pharmacyclics. Vehicle for oral selinexor was 0.6% plasdone PVP K-29/32 and 0.6% poloxamer pluronic F-68. Vehicle for oral ibrutinib was 1% HP-b-CD (Sigma).

**Cell lines**

ABC-DLBCL cell lines RIVA, SUDHL2 and TMD8 and GCB-DLCBCL cell lines OCI-Ly4, SUDHL4, SUDHL5 and Karpas422 were grown in IMDM with 10% fetal calf serum, 100 µg/ml penicillin and streptomycin. OCI-Ly8 (GCB-DLBCL) were grown in equally supplemented RPMI-1640 media. OCI-Ly10 (ABC-DLBCL) cells were grown in IMDM with 20% human plasma, 100 µg/ml penicillin and streptomycin and 50 µM β-mercaptoethanol.

**QRT-PCR**

XPO1 relative expression was determined by quantitative-RT-PCR using the ΔΔCT method and RIVA cells as calibrator.

**Cell proliferation assay and assessment of apoptosis**

Cell proliferation was measured using the CellTiter96^TM^ Cell Proliferation Assay (MTS, Promega). Apoptosis was assessed analyzing the binding of Annexin V-FITC and the incorporation of propidium iodide (PI) by flow cytometry (Bender Medsystems). Annexin V/PI double negative cells were considered viable cells.

**In vivo modeling of PCNSL**

Eight-week-old athymic female mice (NMRI-*Foxn1^nu/nu^* mice; Janvier Labs) were used to develop an orthotopic xenograft model of PCNSL using OCI-Ly10 cells stably transfected with luciferase (Fluc2 gene). For this, 15·10^6^ cells at 1·10^6^ cells/mL were electroporated (960 μF/250V) in the presence of 5pM of pGL4_Luc2_CMV_neo plasmid; 48 hours after electroporation 400μg/ml neomycin was added to culture media. After two weeks of selection, the bioluminescence of cells was analyzed by bioluminescence imaging (BLI) using IVIS^®^ Spectrum system and Living Image software (PerkinElmer). 1·10^5^ cells in 5μl PBS were injected intracerebrally (coordinates: 1mm anterior, 1.8mm lateral right to the bregma and 2.5mm deep from the dura) with a Hamilton syringe with 26-gauge needle at a rate of 1µl/min using a stereotactic platform (Stoelting Just For Mice^TM^). Tumor growth was monitored by bioluminescence imaging (BLI) using an IVIS® Spectrum system (PerkinElmer) twice a week starting at day 4 post-intracerebral injection. For that, mice were anesthetized with isofluorane (1-2%) before intraperitoneal injection of luciferin at a dose of 150mg/kg. Tumoral size was analyzed and quantified using Living Image software (PerkinElmer) and the total photons per second (ph/s) were recorded.

Patient derived xenograft (PDX) model was established by intracerebral implantation of 2·10^5^ human lymphoma cells isolated from brain biopsy by mechanical tissue dissociation in eight-week-old NOD-SCID gamma (NSG) female mice (NOD.Cg-Prkdc^scid^ Il2^tm1Wjl^/SzJ; Charles River Laboratories). Malignant human CD19^+^ cells were sequentially expanded and passaged 3 times *in vivo* until the generation of a stable PDX model. Once a stable PDX in NSG mice was generated, the number of human tumoral cells was sufficient for their implantation in athymic mice for the study of the innate immune response after drug treatments. Thus, 2·10^5^ low-passage CD19^+^ patient-derived tumor cells were stereotactically inoculated into the brain parenquima of eight-week-old athymic female mice as specified above.

For survival experiments, mice were euthanized when end point criteria were met, including neurological symptoms (seizures, circling or hind limb paralysis) or a significant weight loss (>20%).

PDX tumors were confirmed to be negative for Epstein-Barr virus via in situ hybridization (ISH) for EBV-encoded RNA (EBER) (data not shown). ISH was performed on a Ventana BenchMark Ultra autostainer (Ventana Corporation, Tucson, AZ, USA) using EBER probes and the Ventana ISH iVIEW Blue Detection Kit according to manufacturer’s instructions. Mice treated with selinexor were dosed with 5 mg/kg of drug or vehicle via oral gavage three times or twice a week when combined with ibrutinib as detailed in the results section. Ibrutinib was administered daily at 25 mg/kg in drinking water.

**Dissociation of brain tissue and flow cytometry analysis**

Mice brains were collected in cold RPMI-1640 medium immediately after euthanasia and the two hemispheres were separated with a razor blade. One hemisphere was used for immunochemical determinations and the other one was processed for flow cytometry analysis as previously described.^1^ Briefly, brain was dissected and minced through a 100μm cell strainer. Tissue pellets were digested with 1ml 25μg/ml Liberase (Roche), filtered through a 70μm cell strainer and further treated with 10μg/ml DNAse I (Roche). Myelin and cell debris were removed by Percoll (Sigma-Aldrich) density gradient. Cell pellets were further washed and resuspended in 100μl FACS staining buffer (PBS with 1% bovine serum albumin and 0.1% sodium azide (Sigma-Aldrich)). When needed, remaining erythrocytes were lysed using the ACK lysing buffer (Gibco). Cells were blocked with 1μg rat serum IgG per 10^6^ cells (Sigma-Aldrich) for 15min at 4ºC before the incubation with monoclonal antibodies (mAbs) for 20min at 4ºC.

The following mAbs were used for the identification of mouse macrophages and human lymphoma cells: anti-mouse/human CD11b-PerCP Cy5.5 (Clone M1/70), anti-mouse CD19-FITC (Clone 1D3/CD19), anti-mouse CD45-Brilliant Violet 510 (Clone 30-F11), anti-mouse CD206-PE/Cy7 (Clone C068C2), anti-mouse CD279-PE (PD1, Clone 29F.1A12), anti-mouse F4/80-APC/Cy7 (Clone BM8), anti-mouse Gr1-FITC (Clone RB6-8C5) and anti-human CD20-APC (Clone 2H7) were all purchased from Biolegend; anti-mouse CD172a-APC (SIRPα, Clone P84), anti-human CD47-FITC (Clone B6H12) and anti-human CD274-PE (PD-L1, Clone MIH1) were obtained from eBioscience. Dead cells were discarded using the LIVE/DEAD^TM^ Fixable Violet Dead Stain Cell kit (Invitrogen). Mouse tumor-associated macrophages (TAMs) were identified as CD45^+^ Gr1^low/-^ CD11b^+^ F4/80^+^ ;^2^  M1 mouse TAMs as CD206^-^ and M2 mouse TAMs as CD206^+^. All gates were based on fluorescence minus one (FMO) or isotype controls. Flow cytometry was performed in a Navios^TM^ cytometer (Beckman Coulter) and data were analyzed using the FlowJo v10 software (TreeStar).

**Immunohistochemistry**

Antigen retrieval, immunohistochemical (IHC) detection and counterstaining were performed at an Autostainer Link 48 (DAKO) using antibodies against human CD20 (PA5-16701, Thermo Fisher Scientific), human Ki-67 (Clone 30-9, Ventana Medical Systems Inc), mouse F4/80 (Clone SP115, Abcam) and mouse Iba-1 (Clone EPR16588, Abcam). Slides were scanned using NanoZoomer 2.0 HT Digital slide scanner C9600 and visualized using NDP.view 2 (Hamamatsu Photonics K.K).

**Western blot**

Jurkat and Ramos cells treated with the phosphatase-inhibitor pervanadate (3 mM H_2_O_2_/ 1 mM Na_3_VO_4_) for 5 minutes at 37ºC were used as positive controls for phospho-proteins. Whole cell protein extracts were prepared using lysis buffer (20 mM Tris pH 7.4, 1 mM EDTA, 140 mM NaCl, 1% NP-40, 2 mM Na_3_VO_4_ and protease inhibitor cocktail (Sigma-Aldrich)) for 1 hour at 4ºC. Equal amounts of denatured protein were resolved by 10% SDS-PAGE and transferred to Immobilon-P membranes (Millipore). Blocked membranes were incubated overnight at 4ºC with the following primary antibodies: phospho-BTKTyr551/ITKTyr511, BTK (BD Biosciences), phosphoSYK(Tyr352)/phosphoZAP70(Tyr319), phospho-AKTSer473, AKT, phospho-ERK1/2Thr202/Tyr204, ERK1/2 and β-actin (Cell Signaling Technology), SYK (Upstate cell signaling). Images were quantified using Image J software. ^3^ Values of phosphoproteins are expressed as relative to total protein and loading control.

**In vitro culture of human macrophages**

Primary monocytes were isolated from cryopreserved peripheral blood mononuclear cells (PBMC) from healthy donors (HD) by adherence in culture plates. For macrophage differentiation, monocytes were cultured for 5 days in RPMI (Biowest) supplemented with 10% FBS (Gibco) and 50ng/ml M-CSF (StemCell). At day 5, human macrophages were pre-incubated with drugs for 30 minutes and then 10ng/ml IL-10 (Peprotech) was added for 48h to promote M2 differentiation. At day 7, >90% CD14^+^CD16^+^ cells expressed CD206.

Cells were stained with the following mAbs: anti-human CD14-FITC (Clone M5E2) and anti-human CD86-Alexa Fluor 700 (Clone 2331 FUN-1) were purchased from BD; anti-human CD172a-PerCP eFluor710 (SIRPα, Clone 15-414); anti-human PD-L1-PE (Clone MIH5) and anti-human CD206-APC (Clone 19.2) were purchased from eBioscience; anti-human CD16-PE/Cy7 (Clone B73.1), anti-human CD163-Brilliant Violet 605 (Clone GHI/61) and anti-human CD279-A700 (PD1, Clone EH12.2H7) were obtained from Biolegend; anti-human HLA-DR-PC5.5 (Clone Immu-357) was obtained from Beckman Coulter. All gates were based on fluorescence minus one (FMO). Flow cytometry was performed in a Navios^TM^ cytometer (Beckman Coulter) and data were analyzed using the FlowJo v10 software (TreeStar). All gates were based on fluorescence minus one (FMO). Flow cytometry was performed in a Navios^TM^ cytometer (Beckman Coulter) and data were analyzed using the FlowJo v10 software (TreeStar).

**Phagocitosis assay**

The phagocytic capacity of primary macrophages was evaluated using Phagocytosis Assay Kit Red E. coli (Abcam) following manufacturer's instructions.

**IL-10 production**

For IL-10 production, media from cultures of primary M2 macrophages was removed at day 7 and replaced with RPMI free of M-CSF and IL-10. Supernatants were collected after 24h with 1μg/ml LPS (Sigma) and stored at -80ºC. Concentration of IL-10 was determined using ELISA MAX Deluxe Set Human IL-10 (Biolegend) following manufacturer's instructions.

**Statistical analysis**

Results are expressed as the mean ± standard error of the mean (SEM) of at least four independent experiments or subjects. The statistically significant differences between groups were analyzed using the Mann-Whitney test or one or two-way ANOVA, and P < 0.05 was considered significant. Survival curves were generated using the Kaplan and Meier method, and statistically compared by the log-rank test. The synergistic nature of drug interactions was analyzed using isobologram analysis^4^ and the combination index (CI) was calculated according to the Chou–Talalay method.^5^ Analyses were performed using the biostatistics software package SPSS version 22 (IBM, Chicago, IL, USA). Results were graphed with GraphPad Prism 6 software.

**Supplementary references**

1. Pösel C, Möller K, Boltze J, Wagner D-C, Weise G. Isolation and Flow Cytometric Analysis of Immune Cells from the Ischemic Mouse Brain. *J. Vis. Exp. JoVE*. 2016;(108):53658.

2. Gordon SR, Maute RL, Dulken BW, et al. PD-1 expression by tumour-associated macrophages inhibits phagocytosis and tumour immunity. *Nature*. 2017;545(7655):495–499.

3. Schneider CA, Rasband WS, Eliceiri KW. NIH Image to ImageJ: 25 years of image analysis. *Nat. Methods*. 2012;9(7):671–675.

4. Tallarida RJ. An overview of drug combination analysis with isobolograms. *J. Pharmacol. Exp. Ther.* 2006;319(1):1–7.

5. Chou T-C. Drug combination studies and their synergy quantification using the Chou-Talalay method. *Cancer Res.* 2010;70(2):440–446.

**Supplementary figure legends**

**Supplementary figure S1. IHC of representative OCI-Ly10 PCNSL mouse tumors.**

Representative IHC images from brains obtained from 4 nude athymic mice inoculated with OCI-Ly10 cells (24 days after injection). The bar represents 2.5 mm in whole brain images and 250 μm in zoomed in images.

**Supplementary figure S2. Treatment with selinexor and ibrutinib synergizes in DLBCL cell lines**. (A) Phosphorylation of BTK, SYK, AKT and ERK1/2 was analyzed by Western Blot in OCI-Ly10 cells pre-treated with selinexor and/or ibrutinib for 1 hour and stimulated with anti-IgD and anti IgMfor 4 minutes. Quantification of bands is shown relative to cells stimulated with anti-IgD/IgM using total protein and loading control as calibration. (B) Immunoblot showing expression of BTK and β-actin proteins in OCI-Ly10 cells after 12, 24 and 48 hours of treatment with selinexor. Jurkat (T-cell lymphoblastic leukemia) and Ramos (Burkitt’s lymphoma) were used as negative and positive controls respectively for BTK expression. (C) Isobolograms showing synergistic effect of combining selinexor and ibrutinib in vitro. The X axis shows ID50 of ibrutinib while the Y axis shows ID50 of selinexor as single treatments. ID50 of selinexor or ibrutinib alone (square) or when combined with sub-ID50 concentration of the other drug (circle and triangle, for selinexor and ibrutinib respectively) is plotted. PV: pervanadate. S: selinexor. I: ibrutinib.

**Supplementary figure S3. IHC of representative PCNSL PDX tumors.**

Representative IHC images from brains obtained from 2 nude athymic mice inoculated with patient-derived PCNSL cells (18 days after injection). The bar represents 2.5 mm in whole brain images and 250 μm in zoomed in images.

**Supplementary figure S4. Treatment with selinexor and ibrutinib induces downregulation of PD1 and SIRPα in human M2 macrophages.** Human macrophages differentiated from peripheral blood using M-CSF were pre-incubated with drugs for 30 minutes and then 10ng/ml IL-10 was added for 48h to promote M2 differentiation. After 48 hours the following parameters were analyzed: changes in the expression of PD-1 (A), SIRPα (B) and co-expression of both molecules (C) in human M2 macrophages (CD14^+^CD16^+^ CD206^+^) treated with selinexor and/or ibrutinib relative to untreated cells. Changes in phagocytosis (D), CD86 (E), CD163 (F), PD-L1 (G), IL-10 (H), HLA-DR (I) and CD206 (J) in macrophages (CD14^+^CD16^+^) treated with selinexor and/or ibrutinib relative to untreated cells (*P<0.05, **P<0.01, ***P<0.001, Wilcoxon test. Graphs show mean ± SEM).
